# Factors Affecting Recurrent Staphylococcus aureus Bacteremia Among Older Patients in Rural Community Hospitals: A Retrospective Cohort Study

**DOI:** 10.7759/cureus.70120

**Published:** 2024-09-24

**Authors:** Ryuichi Ohta, Chiaki Sano

**Affiliations:** 1 Communiy Care, Unnan City Hospital, Unnan, JPN; 2 Community Medicine Management, Shimane University Faculty of Medicine, Izumo, JPN

**Keywords:** aged, bacteremia, body mass index, recurrence, rural population, staphylococcus aureus

## Abstract

Introduction

Staphylococcus aureus bacteremia (SAB) poses a significant health risk, particularly among adults over 65 years old, due to age-related vulnerabilities and comorbidities. Recurrent SAB is associated with increased morbidity, prolonged hospitalizations, and higher healthcare costs, necessitating the identification of risk factors that contribute to these recurrent infections.

Methods

A retrospective cohort study was conducted at a rural community hospital to identify factors associated with recurrent SAB in older patients. Data were extracted from electronic medical records of patients diagnosed with SAB between April 2016 and December 2023. Multivariate logistic regression was employed to analyze the relationship between recurrent SAB and potential risk factors, including age, sex, BMI, dependency on Japanese long-term health insurance, and comorbidities.

Results

Among 99 patients with SAB, 36 (36.4%) experienced recurrence. Higher BMI was significantly associated with recurrent SAB (OR: 1.15, 95% CI: 1.01-1.31, p = 0.036), while dependency on long-term care was associated with a lower risk of recurrence (OR: 0.20, 95% CI: 0.06-0.64, p = 0.007). Age and sex did not show significant associations with recurrence.

Conclusion

This study identified higher BMI as a risk factor for recurrent SAB in older patients, while dependency on long-term care was protective. These findings highlight the need for targeted management strategies for patients with higher BMI to prevent recurrent SAB. Further research is needed to explore these associations and confirm their relevance in other clinical settings.

## Introduction

Staphylococcus aureus infection is a significant medical concern, particularly among older patients who are more susceptible to severe outcomes due to age-related vulnerabilities and comorbidities [1]. Among the various manifestations of this infection, Staphylococcus aureus bacteremia (SAB) is particularly problematic and associated with high morbidity and mortality rates [2]. The risk is further exacerbated when SAB recurs, leading to a deterioration in the patient's condition and presenting additional challenges in management and treatment [3].

Recurrent SAB significantly complicates patient care, often indicating an incomplete resolution of the initial infection or underlying factors predisposing the patient to repeated episodes [4]. Such recurrences are linked to poorer outcomes, including prolonged hospitalizations, increased healthcare costs, and higher risks of complications like metastatic infections and sepsis [5]. Therefore, identifying and addressing the risk factors associated with recurrent SAB is crucial for improving patient management and outcomes, especially in older populations at heightened risk.

Clarifying the risk of recurrent SAB is essential for timely and effective management of high-risk patients. By understanding these risks, healthcare providers can tailor treatment strategies, implement closer monitoring, and take preventive measures to reduce the likelihood of recurrence [6, 7]. This approach is critical in rural community hospitals, where resources may be limited, and the patient population often includes older individuals with complex healthcare needs [8]. This study aims to elucidate the factors related to recurrent SAB among older patients in a rural community hospital. By focusing on this specific context, the research seeks to provide insights that can enhance clinical practice, improve patient outcomes, and contribute to a better understanding of SAB management in this vulnerable population.

## Materials and methods

This retrospective cohort study explored the factors associated with recurrent SAB in older patients treated at a rural community hospital. Multivariate logistic regression analysis was conducted using data extracted from electronic medical records, with recurrent SAB as the dependent variable. The analysis included covariates such as age, sex, BMI, dependency level based on Japanese long-term care insurance, and existing comorbidities.

Setting

In 2022, Unnan City had a total population of 35,738, comprising 17,231 males and 18,507 females, with 40.27% of the residents aged 65 years or older. A single public hospital served the rural area with 281 beds during the study period. These included 155 acute care beds, 48 general care beds, 30 rehabilitation beds, and 48 chronic care beds. The Department of Family Medicine provided care to internal medicine patients in collaboration with various healthcare professionals [9].

Participants

Participants in the study were selected from patients who sought care at Unnan City Hospital in Unnan, Japan. The inclusion criteria were being over 65 years old and having a positive blood culture for Staphylococcus aureus. Patients under 65 years of age or without a positive blood culture for Staphylococcus aureus were excluded from the study [6,10]. Patient data were gathered from the hospital’s electronic medical records covering the period from April 1, 2016, to December 31, 2023.

Data collection

Recurrent SAB was utilized as the dependent variable in this study. The attending physicians diagnosed recurrent SAB based on their clinical assessment and the concordance of blood culture results [11]. Risk factors for recurrent SAB, identified from previous studies, were assessed as covariates; these data were also extracted from the electronic medical records [6,10]. The covariates included age, sex, body mass index, dependency level based on Japanese long-term care insurance, and existing comorbidities. Patients lacking these specific data were excluded from the study.

Analysis

The normality of continuous variables was assessed before conducting statistical analyses. Depending on the data distribution, parametric data were analyzed using Student's t-test, while nonparametric data were analyzed using the Mann-Whitney U test. Categorical data were examined using the chi-squared test. The following categorical variables were dichotomized for the logistic regression model: sex, dependency on Japanese long-term care insurance (categorized as dependent or not), and recurrent SAB (defined as more than one positive result). Multivariate logistic regression analysis evaluated the association between recurrent SAB and its potential risk factors. These regression models included all variables associated with recurrent SAB, particularly those found to be significant in univariate regression models. All statistical analyses were performed using Easy R, version 1.23 (R Foundation for Statistical Computing, Vienna, Austria) [12]. A p-value of less than 0.05 was considered statistically significant.

Ethical consideration

The Unnan City Hospital Clinical Ethics Committee approved the study protocol (no. 20240010).

## Results

Demographics of the participants

The study included 99 patients diagnosed with SAB at Unnan City Hospital. Of these, 36 (36.4%) experienced recurrent SAB, while 63 (63.6%) had no recurrence. The mean age of the participants was 82.60 years (SD = 11.24), with a slightly older mean age in the non-recurrent group (83.92 years, SD = 11.60) compared to the recurrent group (80.28 years, SD = 10.34). However, this difference was not statistically significant (p = 0.122). Males constituted 53.5% of the overall study population, with similar distributions in both the recurrent (50.0%) and non-recurrent (55.6%) groups (p = 0.677). BMI was significantly associated with recurrent SAB. The mean BMI was statistically higher in the recurrent group (20.20 kg/m², SD = 3.02) than in the non-recurrent group (18.59 kg/m², SD = 3.91), with a p-value of 0.036. Additionally, a significantly lower proportion of patients in the recurrent group were classified as dependent on care under Japanese long-term health insurance (16.7%) compared to the non-recurrent group (46.0%) (p = 0.002). Mortality was also notably different between the groups, with 40.4% of all participants succumbing during the study period. Mortality was significantly higher in the non-recurrent group (54.0%) compared to the recurrent group (16.7%) (p < 0.001). Other comorbidities, including brain stroke, cancer, chronic kidney disease, dementia, diabetes, and heart failure, did not show significant differences between the recurrent and non-recurrent groups (Table 1).

**Table 1:** The demographics of the participants.

Factor	Total	No Recurrence (-)	Recurrence (+)	P-value
n	99	63	36	
Age, years (mean, SD)	82.60 (11.24)	83.92 (11.60)	80.28 (10.34)	0.122
Male Sex (%)	53 (53.5)	35 (55.6)	18 (50.0)	0.677
BMI, kg/m² (mean, SD)	19.17 (3.68)	18.59 (3.91)	20.20 (3.02)	0.036
Dependent condition (%)	35 (35.4)	29 (46.0)	6 (16.7)	0.002
Death (%)	40 (40.4)	34 (54.0)	6 (16.7)	<0.001
Brain stroke (%)	16 (16.2)	11 (17.5)	5 (13.9)	0.78
Cancer (%)	1 (1.0)	1 (1.6)	0 (0.0)	1
Chronic kidney diseases (%)	3 (3.0)	3 (4.8)	0 (0.0)	0.552
Dementia (%)	6 (6.1)	3 (4.8)	3 (8.3)	0.665
Diabetes (%)	28 (28.3)	17 (27.0)	11 (30.6)	0.817
Heart failure (%)	39 (39.4)	25 (39.7)	14 (38.9)	1

Logistic regression model with recurrent SAB infection and related factors

The multivariate logistic regression analysis identified several factors associated with the recurrence of SAB. A higher BMI was associated with an increased likelihood of recurrent SAB, with an OR of 1.15 (95% CI: 1.01-1.31, p = 0.036). Conversely, being classified as dependent on care under Japanese long-term health insurance was significantly associated with a lower risk of recurrence (OR: 0.20, 95% CI: 0.06-0.64, p = 0.007). Age and male sex were not significantly associated with the recurrence of SAB, with ORs of 1.00 (95% CI: 0.95-1.04, p = 0.9) and 0.61 (95% CI: 0.24-1.57, p = 0.31), respectively (Table 2).

**Table 2:** The logistic regression model with recurrent Staphylococcus aureus bacteremia and related factors.

Factor	Odds ratio	95% CI	P-value
Age	1	0.95-1.04	0.9
Male Sex	0.61	0.24-1.57	0.31
BMI	1.15	1.01-1.31	0.036
Dependent condition	0.2	0.06-0.64	0.0071

## Discussion

The findings of this study highlight several important factors associated with the recurrence of SAB among older patients in a rural community hospital setting. Higher BMI was identified as a significant risk factor for recurrent SAB, while dependence on care under Japanese long-term health insurance was associated with a reduced likelihood of recurrence. These findings are consistent with previous studies, though they also offer new insights into the specific context of rural healthcare and the management of older patients.

Several mechanisms could explain the association between higher BMI and recurrent SAB. This study demonstrates the significant relationship between recurrent SAB and higher BMI, underscoring the issues of obesity in SAB. Obesity and higher BMI have been linked to a range of adverse health outcomes, including impaired immune function, chronic inflammation, and increased risk of infections [13, 14]. In the context of SAB, a higher BMI may predispose patients to recurrent infections due to these underlying physiological disturbances [15]. Moreover, obesity can complicate the management of bacterial infections by affecting pharmacokinetics and pharmacodynamics, leading to suboptimal antibiotic concentrations at the infection site [16]. Significantly, in rural contexts where patients with SAB are older, it is challenging to determine the effective dosage of antibiotics for them [17]. This may contribute to an incomplete resolution of the initial infection, thereby increasing the risk of recurrence.

Conversely, the intriguing finding that dependence on care under Japanese long-term health insurance was associated with a lower risk of recurrent SAB warrants further investigation. One possible explanation is that patients classified as dependent on care may receive more comprehensive and consistent care, including better adherence to infection control measures and more frequent medical follow-ups, reducing the likelihood of recurrence [18, 19]. SAB can also develop from external entries such as abrasions and wounds, which may be more common among active older patients than dependent patients [20]. Additionally, older dependent patients might be more closely monitored by healthcare providers and their families, leading to earlier detection and treatment of any signs of infection, thus preventing recurrence [18, 19, 21]. Preventing skin abrasions and wounds in older patients should be prioritized to reduce the recurrence of SAB, and educating patients, caregivers, and healthcare workers can be effective and should be promoted.

The lack of significant associations between age, sex, and recurrent SAB in this study is somewhat unexpected, as older age and male sex have been identified as risk factors for SAB recurrence in other studies [4, 5]. However, the specific characteristics of the study population, such as the homogeneity in age and comorbidities, may have contributed to these findings. These research participants are older than those in previous articles, and most could be included in older population categories of other studies [22, 23]. It is also possible that other unmeasured factors, such as the severity of the initial infection or the presence of undiagnosed comorbidities, could have influenced these results [24]. Previous studies show that male SAB can be critical because of the severity of infection [25, 26]. Future studies can investigate the severity of infection in SAB and the differences in the recurrence of SAB.

This study has several limitations that should be acknowledged. First, the retrospective design inherently limits the ability to establish causal relationships between the identified factors and recurrent SAB. Second, the study was conducted in a single rural community hospital, which may limit the generalizability of the findings to other settings, primarily urban or tertiary care hospitals. The small sample size, especially in the subgroup analyses, may also reduce the statistical power to detect significant associations. Additionally, reliance on electronic medical records for data collection could introduce information bias, particularly if certain variables were not reported or were inaccurately recorded. Finally, the study did not account for certain factors that could influence SAB recurrence, such as the specific strains of Staphylococcus aureus, antibiotic resistance patterns, or the duration and type of antibiotic treatment received.

## Conclusions

This study identified higher BMI as a significant risk factor for recurrent SAB among older patients in a rural community hospital. In contrast, dependence on care under Japanese long-term health insurance was associated with a reduced risk of recurrence. These findings highlight the need for clinicians to consider patient-specific factors, such as BMI and care dependency, in managing SAB among older populations. To reduce the risk of recurrence, targeted interventions such as weight management programs and personalized care plans should be implemented for patients with higher BMI. Moreover, enhancing infection prevention measures and care coordination for those not dependent on long-term care insurance may help mitigate the risks of recurrence.
